# Investigating the Correlation Between Postpartum Management Approaches and Postpartum Depression Symptoms

**DOI:** 10.62641/aep.v53i3.1879

**Published:** 2025-05-05

**Authors:** Jingxia Ying, Yanyan Li, Zhenjuan Li, Qiaoli Tong, Xingmei Li, Xiongying Li

**Affiliations:** ^1^Department of Obstetrics, Yongkang Women and Children’s Health Hospital, 321300 Yongkang, Zhejiang, China; ^2^Department of Group Health Care, Yongkang Women and Children’s Health Hospital, 321300 Yongkang, Zhejiang, China

**Keywords:** postpartum management, postpartum management methods, puerpera, postpartum depression, depressive state

## Abstract

**Background:**

Postpartum depression is a leading public health issue of current international concern. With the change in the concept of postpartum health care, the adjustment of fertility policies, and government support, new postpartum management methods such as maternity matrons and postpartum management centers are becoming increasingly popular. Therefore, the aim of this study is to explore the correlation between postpartum management approaches and postpartum depression symptoms.

**Methods:**

This study recruited 450 postpartum women who gave birth at the Yongkang Women and Children’s Health Hospital, and their data were collected using a convenient sampling method. Out of the total, 150 women received care at the postpartum center of the Yongkang Women and Children’s Health Hospital between June 2022 and February 2024 and were included in the postpartum management centers group, while the other 300 women underwent traditional postpartum care at home, with routine follow-up at the same hospital, and included in the traditional postpartum management group. General information and Edinburgh Postnatal Depression Scale (EPDS) scores were compared between the two groups at 42 days postpartum. Based on EPDS scores, study participants were divided into a postpartum depression symptom group (n = 92) and a non-postpartum depression symptom group (n = 358). Additionally, univariate and multivariate analyses were used to evaluate the factors influencing postpartum depression symptoms.

**Results:**

There were significant differences in maternal education level, family income, and EPDS scores between the postpartum management centers group and the traditional postpartum management group (*p* < 0.05). Univariate analysis identified that family income (*p* < 0.001), employment status (*p* = 0.020), preterm birth (*p* = 0.042), adverse pregnancy history (*p* < 0.001), whether the newborn’s gender meets the family expectation (*p* = 0.005), breast-feeding (*p* < 0.001), adverse postpartum life events (*p* < 0.001), and postpartum management (*p* < 0.001) were associated with postpartum depressive symptoms. Furthermore, lower family income [*p* = 0.013, Odds ratio (OR) = 2.256, 95% Confidence interval (CI) (1.187, 4.287)], adverse pregnancy history [*p* < 0.001, OR = 3.786, 95% CI (1.839, 7.796)], adverse postpartum life events [*p* < 0.001, OR = 11.743, 95% CI (3.669, 37.579)], and traditional postpartum management [*p* < 0.001, OR = 2.842, 95% CI (1.591, 5.075)] were found as risk factors for postpartum depression symptoms. Additionally, neonatal gender conformity with family’s expectations [*p* = 0.010, OR = 0.442, 95% CI (0.239, 0.819)] and breast-feeding [*p* < 0.001, OR = 0.318, 95% CI (0.182, 0.555)] were found as protective factors against postpartum depressive symptoms.

**Conclusion:**

Postpartum depression symptoms are affected by a variety of factors, such as family income, preterm birth, and adverse pregnancy history. Furthermore, postpartum management style is crucial, with women who undergo care in postpartum management centers experiencing a lower risk of postpartum depression symptoms.

## Introduction

Postpartum depression refers to the onset of depression within 12 months following delivery, with low mood as the core feature. It mainly manifests as feelings of sadness, emptiness, or irritability and is accompanied by severe cognitive and physical symptoms, which is a major public health problem of current international concern [1, 2, 3]. This condition adversely impacts maternal energy and physical recovery, affects family relationships, and increases the risk of alcohol or drug abuse. In severe cases, it may result in self-injury or suicide [4]. Previous studies have shown that postpartum depression may impair the intellectual, emotional, and personality development of offspring, increasing the risk of attention deficit hyperactivity disorder (ADHD) in children [5, 6]. Therefore, maternal mental health is crucial and needs significant attention. Despite its clinical significance, the maternal mental health issue has not received adequate focus, with studies revealing a relatively high postpartum depression rate among Chinese women [7, 8]. Since 2014, China has gradually relaxed its policies to encourage childbirth. This investigation holds practical significance for implementing these policies and improving the fertility rate.

The postpartum period refers to the time from the delivery of the placenta to the recovery of all maternal organs, except for the mammary gland, to the pre-pregnancy level, usually lasting 6–8 weeks [9]. During this period, mothers experience significant physical, psychological, and social changes, along with the need for complex parenting tasks [10]. The psychological condition of postpartum women is relatively vulnerable, and several factors like economic pressures and stressful events elevate the risk of postpartum depression and other mental health problems [11, 12]. In China and some Asian countries, postpartum management is a unique custom. With the advancing concept of postpartum health care, adjustments to fertility policies, and government support, new postpartum management strategies, such as postpartum matrons and postpartum management centers are becoming increasingly popular. Although numerous studies have explored the factors affecting postpartum depression [13, 14, 15, 16], there is a lack of investigations on the correlation between postpartum management approaches and postpartum depression.

To address the gap, this study collected general information on postpartum women who gave birth at the Yongkang Women and Children’s Health Hospital, comprehensively screened for depressive symptoms and explored the correlation between different postpartum management approaches and postpartum depression. The aim is to improve the psychological health status of postpartum women, provide scientifically based postpartum care and educational guidance, and play a positive role in creating a supportive environment for childbirth.

## Methods

### Study Participants

This study recruited 450 postpartum women who gave birth at the Yongkang Women and Children’s Health Hospital, and their data were collected using a convenient sampling method. Out of the total, 150 postpartum women who received postpartum care at the postpartum center of the Yongkang Women and Children’s Health Hospital between June 2022 and February 2024 were included in the postpartum management centers group. Additionally, 300 postpartum women who underwent the traditional postpartum management method at home, with routine follow-up at the same hospital, were included in the traditional postpartum management group.

The inclusion criteria were as follows: (1) Patients voluntarily participating in this study, (2) those residing within the healthcare jurisdiction of our city, (3) those willing to cooperate with postpartum visits and the routine 42-day postpartum follow-up, and (4) maternal education level of primary school or above, with clear consciousness and normal understanding ability. The exclusion criteria were as follows: Individuals with a history of mental illness, congenital disabilities, and severe postpartum complications or other obstetric diseases.

### Experimental Design

All investigators involved in this study received standard training. Before distributing the questionnaire, researchers informed participants about the purpose, significance, content, precautions, and expected survey duration. Furthermore, investigators emphasized the voluntary nature of the participation and ensured that the study would not involve any privacy related questions to obtain their support. Participants were instructed to complete the questionnaire independently. For those unable to fill out the questionnaire due to illness or educational limitations, researchers performed one-on-one interviews and recorded their answers. All questionnaires were collected and verified by a designated investigator.

Participants completed two questionnaires: (1) General information form: This form was designed to collect data regarding the participant’s age, mode of delivery, nationality, household registration, education level, family income, employment status, type of pregnancy, multiple pregnancies, instances of premature birth or congenital disabilities, history of adverse pregnancies, whether the pregnancy was planned, whether the newborn’s gender meets family’s expectations, feeding mode, adverse postpartum life events experienced in the 42-day postpartum period (e.g., marital problems, discord/suspicion or betrayal, serious illness or death of a close one, accidents, natural disasters, and financial issues), and the postpartum management strategy used. (2) Postpartum depression assessment: The Edinburgh Postnatal Depression Scale (EPDS) [17] was applied for screening during the routine 42-day follow-up visits at the postpartum clinic. This scale comprises 10 items, each scored on a scale ranging from 0 to 3 points based on symptom frequency, with a total score of 30 points. An EPDS score of ≥9 was used as the threshold for developing postpartum depression. Individuals scoring ≥9 were referred to a psychological counseling clinic or psychiatric department for further assessment or intervention. Those exhibiting suicidal thoughts or erratic behavior were immediately referred to a psychiatric hospital.

After completion, the questionnaires were collected on-site and reviewed by two researchers to ensure accuracy, eliminating those that were incomplete, logically inconsistent, or had invalid or regular responses.

### Statistical Analysis

Statistical analysis was conducted using SPSS v23.0 (IBM SPSS Corp.; Armonk, NY, USA). Categorical variables were analyzed using *Chi-Squared* or *Chi-Squared correction* or *Fisher’s exact test*. Continuous data (age and EPDS score) was evaluated for normality using the Shapiro-Wilk normality test. Normally distributed data (age) were expressed as mean ± standard deviation ($\bar{x}$ ± s) and analyzed using an independent sample *t*-test. Moreover, non-normally distributed data (EPDS score) were represented as median (minimum, maximum) and analyzed using the Mann-Whitney U test. Additionally, univariate and multiple logistic regression analyses were performed to identify factors influencing depressive symptoms in postpartum women. A *p*-value of <0.05 was considered statistically significant.

## Results

### Comparison of General Information and EPDS Scores Between Different Postpartum Management Groups

There were no significant differences between the postpartum management centers group and the traditional postpartum management group regarding maternal age, mode of delivery, nationality, household registration, employment status, natural pregnancy, multiple pregnancies, preterm birth, congenital disabilities, history of adverse pregnancies, whether the pregnancy was planned, whether the newborn’s gender meets family’s expectations, breast-feeding, and adverse postpartum life events (*p* > 0.05). However, education level and family income were significantly higher in the postpartum management center group than those in traditional postpartum management group (*p* < 0.05); furthermore, the EPDS score was significantly lower than that of the traditional postpartum management group (*p* < 0.05, Table 1).

**Table 1.** **Comparison of general information and EPDS scores between the two postpartum management groups**.

Variables		Postpartum management centers group (n = 150)	Traditional postpartum management group (n = 300)	*t/Z/χ^2^*	*p*-value
Age (years)		30.91 ± 4.65	30.40 ± 5.62	0.960	0.338
Mode of delivery [n (%)]				1.811	0.178
	Cesarean section	56 (37.33)	93 (31.00)		
	Spontaneous labor	94 (62.67)	207 (69.00)		
Nationality [n (%)]				0.933	0.334
	The Han nationality	143 (95.33)	279 (93.00)		
	Other	7 (4.67)	21 (7.00)		
Household registration [n (%)]				1.602	0.206
	Local	121 (80.67)	256 (85.33)		
	Other places	29 (19.33)	44 (14.67)		
Education level [n (%)]				256.861	<0.001
	Primary school	3 (2.00)	21 (7.00)		
	Middle school	37 (24.67)	183 (61.00)		
	Junior college	98 (65.33)	2 (0.67)		
	Undergraduate course	4 (2.67)	87 (29.00)		
	Postgraduate	8 (5.33)	7 (2.33)		
Family income [n (%)]				174.080	<0.001
	<50,000 CNY	1 (0.67)	113 (37.67)		
	50,000∼100,000 CNY	6 (4.00)	91 (30.00)		
	100,000∼300,000 CNY	62 (41.33)	63 (21.00)		
	>300,000 CNY	81 (54.00)	33 (11.00)		
Employment status [n (%)]				0.272	0.602
	Unemployed	19 (12.67)	33 (11.00)		
	Employed	131 (87.33)	267 (89.00)		
Natural pregnancy [n (%)]				2.344	0.126
	Yes	141 (94.00)	291 (97.00)		
	No	9 (6.00)	9 (3.00)		
Multiple pregnancy [n (%)]				0.010	0.921
	Yes	5 (3.33)	8 (2.67)		
	No	145 (96.67)	292 (97.33)		
Preterm birth [n (%)]				0.810	0.368
	Yes	7 (4.67)	9 (3.00)		
	No	143 (95.33)	291 (97.00)		
Congenital disabilities [n (%)]					0.767
	Yes	1 (0.67)	1 (0.33)		
	No	149 (99.33)	299 (99.67)		
History of adverse pregnancy [n (%)]				0.630	0.427
	Yes	12 (8.00)	31 (10.33)		
	No	138 (92.00)	269 (89.67)		
Whether the pregnancy was planned [n (%)]				1.409	0.235
	Yes	126 (84.00)	238 (79.33)		
	No	24 (16.00)	62 (20.67)		
Whether the gender of the newborn meets family’s expectations [n (%)]				0.007	0.931
	Yes	123 (82.00)	245 (81.67)		
	No	27 (18.00)	55 (18.33)		
Breast-feeding [n (%)]				0.894	0.344
	Yes	119 (79.33)	226 (75.33)		
	No	31 (20.67)	74 (24.67)		
Adverse postpartum life events [n (%)]				0.260	0.610
	Yes	5 (3.33)	13 (4.33)		
	No	145 (96.67)	287 (95.67)		
EPDS score (points)		5 (3, 7)	6 (3, 9)	2.821	0.005

Notes: EPDS, Edinburgh Postnatal Depression Scale. 1 CNY ≈ 0.13822 USD.

### Univariate Analysis of Postpartum Depression Symptoms and Non-postpartum Depression Symptom Groups

Based on the EPDS scores, 450 participants were divided into a postpartum depression symptom group (n = 92) and a non-postpartum depression symptom group (n = 358). Univariate analysis indicated that family income (*p* < 0.001), employment status (*p* = 0.020), preterm birth (*p* = 0.042), history of adverse pregnancy (*p* < 0.001), whether the gender of the newborn meets family’s expectations (*p* = 0.005), breast-feeding (*p* < 0.001), adverse postpartum life events (*p* < 0.001), and postpartum management strategy (*p* < 0.001) were associated with postpartum depression symptoms (*p* < 0.05, Table 2).

**Table 2.** **Univariate analysis of postpartum depression symptoms and non-postpartum depression symptom groups**.

Variables		Postpartum depression symptoms group (n = 92)	Non-postpartum depression symptom group (n = 358)	*t/χ^2^*	*p*-value
Age (years)		30.38 ± 5.57	30.61 ± 5.25	0.377	0.707
Mode of delivery [n (%)]				2.567	0.108
	Cesarean section	24 (26.09)	125 (34.92)		
	Spontaneous labor	68 (73.91)	233 (65.08)		
Nationality [n (%)]				0.018	0.894
	The Han nationality	86 (93.48)	336 (93.85)		
	Other	6 (6.52)	22 (6.15)		
Household registration [n (%)]				0.860	0.354
	Local	80 (86.96)	297 (82.96)		
	Other places	12 (13.04)	61 (17.04)		
Education level [n (%)]				8.196	0.085
	Primary school	6 (6.52)	18 (5.03)		
	Middle school	54 (58.70)	166 (46.37)		
	Junior college	2 (2.17)	4 (1.12)		
	Undergraduate course	26 (28.26)	159 (44.41)		
	Postgraduate	4 (4.35)	11 (3.07)		
Family income [n (%)]				25.098	<0.001
	<50,000 CNY	41 (44.57)	73 (20.39)		
	50,000∼100,000 CNY	20 (21.74)	77 (21.51)		
	100,000∼300,000 CNY	16 (17.39)	109 (30.45)		
	>300,000 CNY	15 (16.30)	99 (27.65)		
Employment status [n (%)]				5.423	0.020
	Unemployed	17 (18.48)	35 (9.78)		
	Employed	75 (81.52)	323 (90.22)		
Natural pregnancy [n (%)]				0.239	0.625
	Yes	87 (94.57)	345 (96.37)		
	No	5 (5.43)	13 (3.63)		
Multiple pregnancy [n (%)]				0.012	0.912
	Yes	3 (3.26)	10 (2.79)		
	No	89 (96.74)	348 (97.21)		
Preterm birth [n (%)]				4.154	0.042
	Yes	7 (7.61)	9 (2.51)		
	No	85 (92.39)	349 (97.49)		
Congenital disabilities [n (%)]				0.026	0.873
	Yes	1 (1.09)	1 (0.28)		
	No	91 (98.91)	357 (99.72)		
History of adverse pregnancy [n (%)]				16.476	<0.001
	Yes	19 (20.65)	24 (6.70)		
	No	73 (79.35)	334 (93.30)		
Whether the pregnancy was planned [n (%)]				1.032	0.310
	Yes	71 (77.17)	293 (81.84)		
	No	21 (22.83)	65 (18.16)		
Whether the gender of the newborn meets family’s expectations [n (%)]				7.820	0.005
	Yes	66 (71.74)	302 (84.36)		
	No	26 (28.26)	56 (15.64)		
Breast-feeding [n (%)]				16.132	<0.001
	Yes	56 (60.87)	289 (80.73)		
	No	36 (39.13)	69 (19.27)		
Adverse postpartum life events [n (%)]				21.758	<0.001
	Yes	12 (13.04)	6 (1.68)		
	No	80 (86.96)	352 (98.32)		
Postpartum management method [n (%)]				13.226	<0.001
	Postpartum management center	16 (17.39)	134 (37.43)		
	Traditional postpartum management	76 (82.61)	224 (62.57)		

Notes: EPDS, Edinburgh Postnatal Depression Scale; Postpartum depression symptoms group, EPDS score ≥9 points; non-postpartum depression symptoms group, EPDS score <9 points. 1 CNY ≈ 0.13822 USD.

### Multiple Logistic Regression Analysis of Postpartum Depression Symptoms

Multiple logistic regression was performed with postpartum depression symptoms as the dependent variable, and family income (100,000~300,000 CNY and >300,000 CNY = 0, <50,000 CNY and 50,000–100,000 CNY = 1. 1 CNY ≈ 0.13822 USD), employment status (employed = 0, unemployed = 1), preterm birth (no = 0, yes = 1), history of adverse pregnancy (no = 0, yes = 1), whether the newborn’s gender meets family expectations (no = 0, yes = 1), breast-feeding (no = 0, yes = 1), adverse postpartum life events (no = 0, yes = 1), postpartum management approaches (postpartum management center = 0, traditional postpartum management = 1) as independent variables.

The analysis identified low family income [*p* = 0.013, Odds ratio (OR) = 2.256, 95% Confidence interval (CI) (1.187, 4.287)], history of adverse pregnancy [*p* < 0.001, OR = 3.786, 95% CI (1.839, 7.796)], adverse postpartum life events [*p* < 0.001, OR = 11.743, 95% CI (3.669, 37.579)], and traditional postpartum management method [*p* < 0.001, OR = 2.842, 95% CI (1.591, 5.075)] as significant risk factors for postpartum depression symptoms. In contrast, neonatal gender conformity with family expectations [*p* = 0.010, OR = 0.442, 95% CI (0.239, 0.819)] and breast-feeding [*p* < 0.001, OR = 0.318, 95% CI (0.182, 0.555)] were found as protective factors against postpartum depressive symptoms (Table 3).

**Table 3.** **Multiple logistic regression analysis of postpartum depression symptoms**.

Variables	B	Standard error	Wald	*p*-value	Odds ratio (OR)	95% Confidence interval (CI)	
						Lower	Upper
Family income	0.814	0.328	6.167	0.013	2.256	1.187	4.287
Employment status	0.672	0.376	3.206	0.073	1.959	0.938	4.089
Preterm birth	1.065	0.600	3.150	0.076	2.901	0.895	9.403
History of adverse pregnancy	1.331	0.369	13.053	<0.001	3.786	1.839	7.796
Whether the gender of the newborn meets the expectations of the family	–0.815	0.314	6.725	0.010	0.442	0.239	0.819
Breast-feeding	–1.145	0.284	16.257	<0.001	0.318	0.182	0.555
Adverse postpartum life events	2.463	0.593	17.226	<0.001	11.743	3.669	37.579
Traditional postpartum management method	1.044	0.296	12.452	<0.001	2.842	1.591	5.075
Constant	–1.268	0.446	8.070	0.005	0.281		

## Discussion

This study conducted a questionnaire survey on 450 postpartum women 42 days after delivery, with 92 individuals (over one-fifth) experiencing postpartum depression (EPDS score ≥9). The findings from this study highlight the significance of comprehensively exploring the factors impacting postpartum depression and implementing appropriate intervention measures.

Our findings revealed that the postpartum management centers group had higher levels of maternal education and household income compared to the traditional postpartum management group. This can be due to two situations: Firstly, the higher costs associated with postpartum management centers make them unaffordable for lower-income families. Secondly, mothers with lower levels of education often adhere to traditional approaches to postpartum management, believing it naturally occurs at home or with their mother-in-law. These individuals tend to be less receptive to “new ideas and concepts”, show lower independence, and mostly follow the guidance of their elders.

This study combined univariate and multivariate analysis and revealed family income, history of adverse pregnancy, whether the gender of the newborn meets family’s expectations, breast-feeding, adverse postpartum life events, and postpartum management method as independent risk factors for postpartum depression symptoms. Despite the postpartum management method, other factors have been widely reported in previous studies. For example, Wang *et al*. [18] reviewed 565 studies from over 80 countries, indicating a strong association between breast-feeding, economic difficulties, life stress, and postpartum depression. Similarly, van der Zee-van den Berg *et al*. [19] investigated 1406 postpartum women and observed adverse postpartum life events are risk factors for postpartum depression and anxiety. Furthermore, Rong *et al*. [20] conducted logistic regression analysis on singleton pregnant women and found that whether the newborn’s gender met family expectations accounted for about 15% of the postpartum depression risk. Ye *et al*. [21] found through meta-analysis that women who gave birth to girls had a higher risk of postpartum depression compared to those who gave birth to boys. These findings align closely with our results.

Therefore, we primarily focus on the effect of postpartum management method on postpartum depression. Among the 92 mothers who may be suffering from postpartum depression, 82.61% were following traditional postpartum management method. The outcomes may be attributed to several factors: (1) Traditional postpartum management: It is performed at home, where caregiving is usually provided by elders such as mother or mother-in-law take. However, age, personality, and educational level differences often lead to conflicting parenting concepts. For example, some elders believe that repurposing old clothes as diapers is economical and non-irritating to the skin. These differences can lead to conflicts and emotional distress for the mothers. In contrast, postpartum management centers offer professional care, providing relevant training to improve mothers’ parenting knowledge and skills. (2) Emotional support: During the postpartum period, the husbands and elders may pay more attention to the baby, often resulting in reduced attention to the mothers’ emotional needs. This neglect can result in emotional distress in mothers. Conversely, postpartum management centers focus on the mothers’ psychological well-being, providing professional psychological support. (3) Nutrition and dietary needs: Sometimes postpartum dietary needs are neglected, resulting in nutritional imbalance. For instance, some mothers-in-law emphasize excessive consumption of eggs, chicken soup, crucian carp soup, or pig trotter soup, leading to overnutrition. However, postpartum management centers provide food prepared by professional nutritionists, balancing taste, nutrition, and other needs, which improves breast-feeding and overall maternal health. (4) Clothing for postpartum women: Family elders usually insist that mothers wear tight clothing, add an extra layer, wear hats, and even tightly wrap the cuffs and pants. They may avoid washing hair or showering, and neglect indoor ventilation to prevent the mother from catching a cold. However, due to the increased metabolism after childbirth, excessive clothing can cause sweating and discomfort. Conversely, postpartum management centers provide a constant environment with optimal temperature and humidity, suggesting to mothers that regular, comfortable clothing of appropriate thickness is sufficient. (5) Postpartum women need a quiet and comfortable environment, which is usually disrupted at home by frequent visits from family and friends. Generally, the environment at home is relatively noisy, increasing restlessness for postpartum women. Zhao and Zhang [22] reported that sleep disruption and poor sleep quality are the risk factors associated with postpartum depression. (6) Conversely, postpartum management centers provide professional postpartum recovery services, assisting mothers with pelvic floor muscles and rectus abdominis muscle repair, as well as weight management, alleviating feelings of inferiority and anxiety. Garcia *et al*. [23] observed low self-esteem as a risk factor for postpartum depression in military spouses. (7) Some mothers-in-law may interfere the daily life of couples, leading to conflicts and reduced social support for women. Numerous studies have highlighted the impact of poor mother-in-law and daughter-in-law relationships as factors contributing to postpartum depression [24, 25, 26, 27]. Chung *et al*. [28] investigated 783 women who gave birth in Pakistan and found that maternal care by mothers-in-law caring was associated with an increased risk of perinatal depression, supporting similar conclusion. However, Peng *et al*. [29] did not find a correlation between the primary caregiver and postpartum depression risk in their investigation of 1325 pregnant women, focusing on home postpartum management without comparing it with the postpartum management centers. Liu *et al*. [30] found that low social support is also an independent influencing factor leading to postpartum depression. Hence, our study is the first to explore the correlation between different postpartum management approaches and postpartum depression, addressing a crucial gap in this research area.

However, it is worth noting that although this study confirms a lower postpartum depression incidence among women in postpartum management centers, these centers may present some challenges. The unfamiliar environment and limited freedom may potentially impact family relationships. Additionally, the professional level and service attitude of some staff in the postpartum management centers need to be improved, and the price can be relatively high. Therefore, when selecting a postpartum management method, women should consider their situations and preferences.

Despite promising outcomes, our study has certain limitations which need to be addressed: (1) Although this survey included 450 postpartum women, the sample size remains relatively small, which can impact the generalizability of the results. (2) In the general information questionnaires, some individuals did not provide accurate details about their education levels and household income, which may affect the accuracy of the results. (3) The EPDS is a self-assessment scale, and responses may be influenced by subjective factors, such as the likelihood of intentional manipulation to align with individual expectations or assumptions. (4) Due to family income and other factors, fewer women in this study underwent care at the postpartum management centers, while a larger proportion of women chose traditional postpartum management methods, leading to a significant variation in sample size between the two groups, which may lead to bias in statistical analysis. Therefore, future research should aim to increase the sample size and follow objective quantitative measures to improve the accuracy and reliability of the findings.

## Conclusion

Postpartum depression symptoms are affected by various factors, such as family income, preterm birth, and adverse pregnancy history. Additionally, postpartum management style plays a crucial role, with those who attend postpartum management center experiencing a reduced risk of postpartum depression symptoms.

## Availability of Data and Materials

The data used to support the findings of this study are included within the article, and during the present study are available from the corresponding author on reasonable request.
